# Supplementary material for: Loss of Protein Function Causing Severe Phenotypes of Female-Restricted Wieacker Wolff Syndrome due to a Novel Nonsense Mutation in the ZC4H2 Gene
Source: Genes (Basel). 2022 Aug 29;13(9):1558. doi: 10.3390/genes13091558 (PMC9498907; doi:10.3390/genes13091558)
Supplement: Supplementary file 1 [file genes-13-01558-s001.zip › genes-1864667-supplementary figures.pdf]

Supplementary Figures

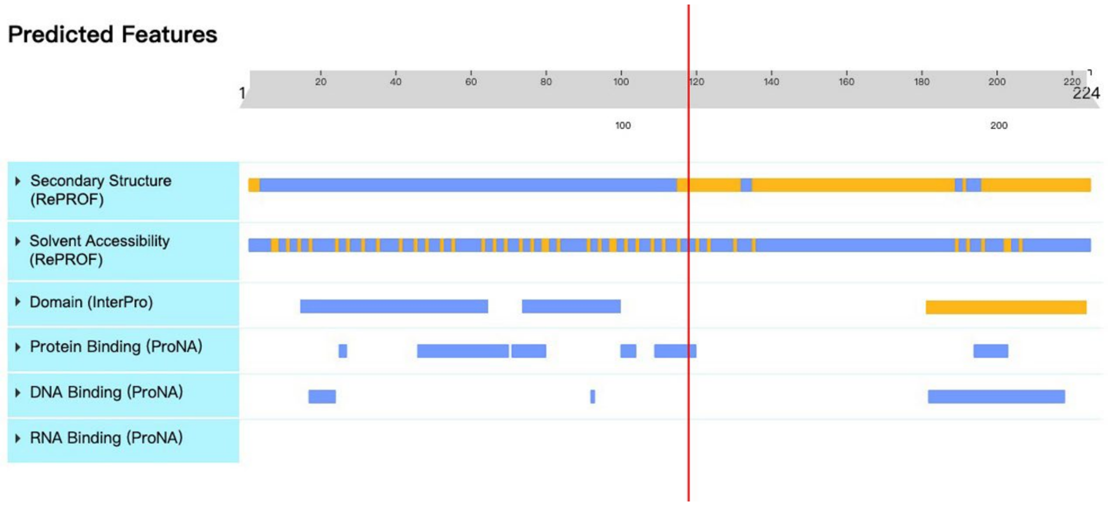

Figure S1. Secondary structure analysis and solvent accessibility analysis were performed using the PredictProtein server. Blue stripes in the secondary structure line denote helix, and yellow represent other structures. Blue stripes in the solvent accessibility line indicate the residues were exposed to solvents, and yellows mean not. Domain analysis was performed using the InterPro, in which two blue stripes were two coil domains, and the yellow one meant C-terminal zinc-finger domain. Binding predictions were performed using the ProNA2020, in which blue stripes were the binding residues.

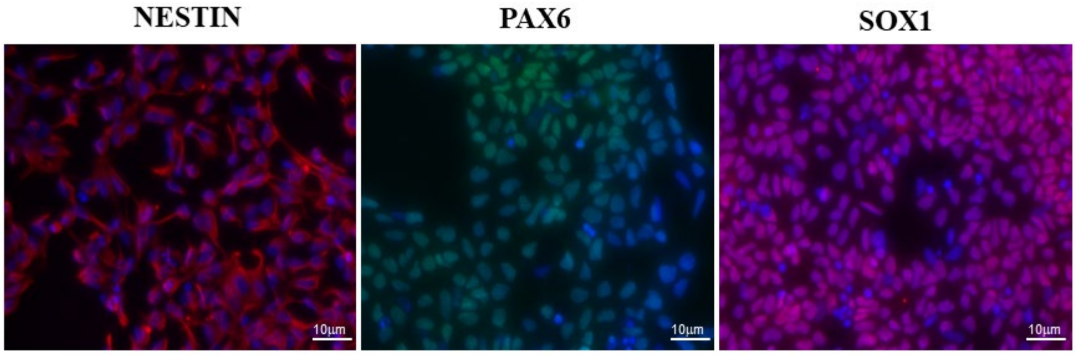

Figure S2. Immunofluorescence images of the NSCs markers including NESTIN, PAX6 and SOX1, were taken using a fluorescence microscope. Scale bars = 10 μm.

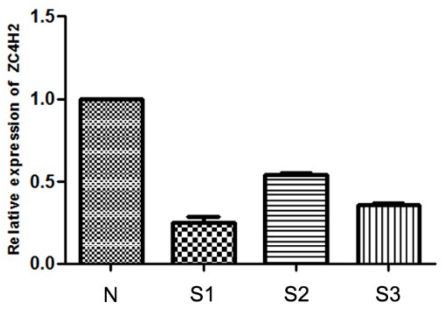

Figure S3. The NSCs were infected with three different lentivirus of shRNA targeting *ZC4H2*. Relative expression levels of *ZC4H2* were identified using RT-qPCR. N: NSCs infected with negative control lentivirus; S1: NSCs infected with lentivirus ZC4H2-shRNA-1; S2: NSCs infected with lentivirus ZC4H2-shRNA-2; S3: NSCs infected with lentivirus ZC4H2-shRNA-3. The expression levels of *ZC4H2* in three different knockdown NSCs reached only 25% (0.25±0.06), 54% (0.54±0.013) and 36% (0.36±0.013) of that in the negative control NSCs, respectively.

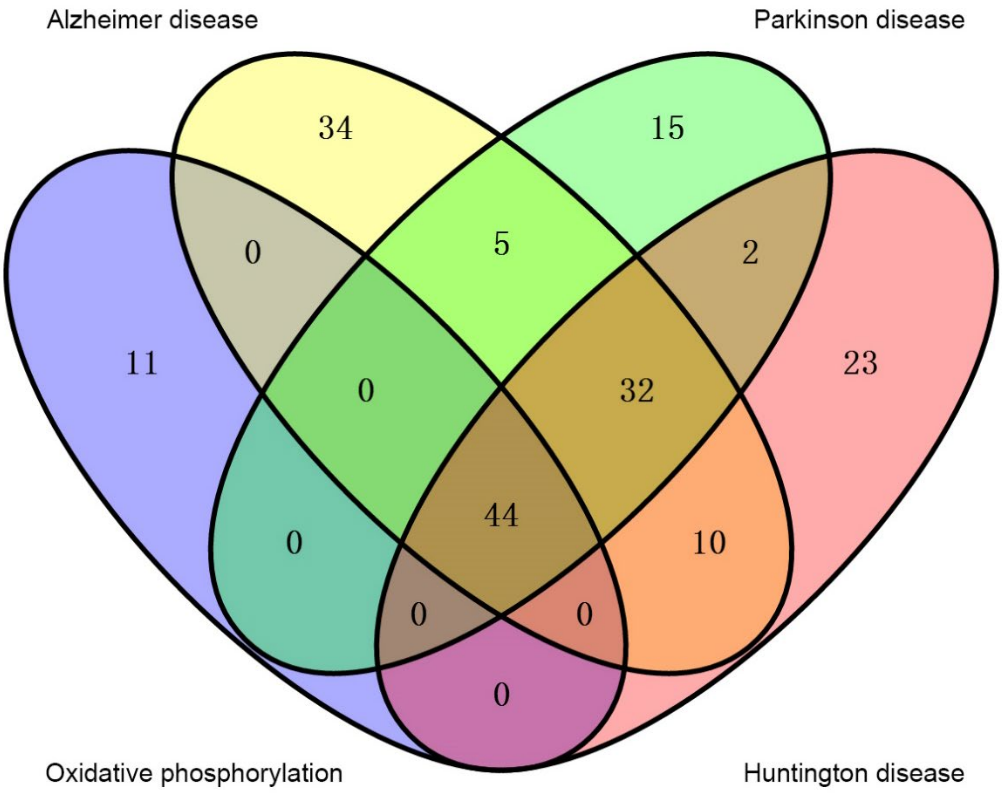

Figure S4. Venny diagram analysis of DEGs. The yellow circle represented 125 DEGs related to Alzheimer disease; The green circle represented 98 DEGs associated with Parkinson disease; The blue circle represented 55 DEGs associated with Oxidative phosphorylation; The red circle represented 111 DEGs associated with Huntington disease. A total of 44 genes were involved in these signaling pathways.
