# Supplementary material for: Loss of Protein Function Causing Severe Phenotypes of Female-Restricted Wieacker Wolff Syndrome due to a Novel Nonsense Mutation in the ZC4H2 Gene
Source: Genes (Basel). 2022 Aug 29;13(9):1558. doi: 10.3390/genes13091558 (PMC9498907; doi:10.3390/genes13091558)
Supplement: Supplementary file 1 [file genes-13-01558-s001.zip › genes-1864667-supplementary tables.pdf]

Supplementary Tables

Table S1. The sequences of the shRNA target the human *ZC4H2* gene.

NO.	Accession	Target Seq	GC%
ZC4H2-shRNA-1 (S1)	NM_018684	gcACCTGAAGGAATACAAGCA	42.11%
ZC4H2-shRNA-2 (S2)	NM_018684	ttCACCGGAATGCACCTATAT	47.37%
ZC4H2-shRNA-3 (S3)	NM_018684	acAATCTGAGAATGACCTAAA	31.58%

Table S2. Sequences of TaqMan® probes for the mitochondria genes.

Gene	Assay ID	Fluorophore
GAPDH	Hs03929097_g1	VIC-MGB
MT-COX1	Hs02596864_g1	FAM-MGB
MT-COX3	Hs02596866_g1	FAM-MGB
MT-Cytb	Hs02596867_s1	FAM-MGB

Table S3. Sequences of specific primers for oxidative phosphorylation complex genes.

Primers	Sequences
COX6A1-F	5′-AGTTGGTGTGTCCTCGGTTTC-3′
COX6A1-R	5′-GTGAGAGTCTTCCACATGCGA-3′
COX6B1-F	5′-CTACAAGACCGCCCCTTTTGA-3′
COX6B1-R	5′-GCAGAGGGACTGGTACACAC-3′
COX8A-F	5′-GCCAAGATCCATTCGTTGCC-3′
COX8A-R	5′-CTCTGGCCTCCTGTAGGTCT-3′
NDUFA1-F	5′-GCGTACATCCACAGGTTCACT-3′
NDUFA1-R	5′-GCGCCTATCTCTTTCCATCAGA-3′
NDUFA3-F	5′-GGGGCCTCGCTGTAATTCTG-3′
NDUFA3-R	5′-GACGGGCACTGGGTAGTTG-3′
NDUFB3-F	5′-GCTGGCTGCAAAAGGGCTA-3′
NDUFB3-R	5′-CTCCTACAGCTACCACAAATGC-3′

Table S4. Enriched KEGG pathways from the differentially expressed genes.

ID	Description	GeneRatio	pvalue	p.adjust	qvalue	geneName
hsa00190	Oxidative phosphorylation	55/1653	3.10E-08	1.27E-06	1.05E-06	UQCRH,COX8A,COX1,COX6A1,NDUFA1,ND4L,ND3,COX6B1,ATP6V0A1,ATP5MF,UQCR10,SDHB,ATP5F1D,PPA1,UQCRC2,COX2,UQCRB,ATP5MC3,NDUFA3,NDUFB7,ATP5PF,COX5A,SDHA,ATP6V1E1,ATP5MC2,CYC1,ATP6V1C1,COX5B,ATP6V1G1,NDUFB1,CYCS,ATP6V1F,NDUFC2,NDUFC1,ATP5F1C,NDUFB3,NDUFS6,NDUFV1,NDUFS1,ND2,NDUFB4,NDUFS7,SDHC,ND4,NDUFAB1,ATP5MC1,ATP5ME,COX6C,ATP6V0D1,ATP6V1H,NDUFB10,ATP5PD,CYTB,ATP6V1D,COX4I1
hsa05010	Alzheimer disease	125/1653	6.45E-09	3.02E-07	2.50E-07	KIF5C,WNT7B,TUBB4B,UQCRH,AXIN2,COX8A,COX1,SLC25A6,CTNNB1,COX6A1,NDUFA1,RAF1,IRS1,TUBB,ND4L,FZD10,ND3,COX6B1,FZD2,SEM1,MAP2K2,PSMB7,APC2,UQCR10,PLCB3,SDHB,ATP5F1D,CSNK1E,DVL1,FAS,PSENEN,MAP2K7,UQCRC2,PSMD2,MAPK10,TUBB2A,DVL2,PIK3C3,COX2,PSMA7,TUBA1B,UQCRB,PSMC3,ATP5MC3,NDUFA3,NDUFB7,ATP5PF,COX5A,SDHA,ATP5MC2,PSMD3,CYC1,SLC39A9,CSNK2A3,COX5B,WNT5A,PSMC1,MAPK8,CALM3,ULK2,FZD9,NDUFB1,PSMD4,WNT7A,CYCS,FZD1,PSMD11,NDUFC2,NDUFC1,TUBB6,ATP5F1C,NDUFB3,VDAC1,NDUFS6,NDUFV1,PSEN1,NDUFS1,ND2,FZD3,NDUFB4,NDUFS7,SDHC,NCSTN,APAF1,TUBB3,BAD,ATG13,FZD5,WNT9A,CAPN1,SLC25A5,PSMA3,PSMD14,ATG2B,ND4,EIF2S1,MAPK9,RTN4,NDUFAB1,RB1CC1,ATP5MC1,AXIN1,PSMA5,EIF2AK2,PSMB5,SLC11A2,COX6C,MAP2K1,NRBF2,KLC2,PSMD8,MAPK1,SLC39A14,NDUFB10,GNAQ,ATP5PD,PSMC5,APH1B,CYTB,TUBA1A,TUBB4A,CDK5R1,PLCB4,GSK3B,COX4I1
hsa05012	Parkinson disease	98/1653	2.07E-10	1.36E-08	1.13E-08	KIF5C,TUBB4B,UQCRH,COX8A,COX1,SLC25A6,COX6A1,NDUFA1,TUBB,ND4L,UBA52,ND3,COX6B1,PARK7,SEM1,PSMB7,UQCR10,PRKACA,SDHB,ATP5F1D,UQCRC2,PSMD2,HSPA5,GNAI2,MAPK10,TUBB2A,COX2,PSMA7,TUBA1B,SOD1,UQCRB,RPS27A,PSMC3,ATP5MC3,NDUFA3,NDUFB7,ATP5PF,COX5A,SDHA,DAXX,ATP5MC2,PSMD3,UBE2J2,CYC1,SLC39A9,COX5B,PSMC1,MAPK8,CALM3,UBC,NDUFB1,UBE2G2,PSMD4,CYCS,PLCG1,PSMD11,BAX,NDUFC2,NDUFC1,TUBB6,ATP5F1C,NDUFB3,VDAC1,NDUFS6,NDUFV1,TH,NDUFS1,ND2,NDUFB4,NDUFS7,SDHC,APAF1,TUBB3,GNAS,SLC25A5,PSMA3,PSMD14,ND4,EIF2S1,MAPK9,NDUFAB1,ATP5MC1,PSMA5,PSMB5,SLC11A2,COX6C,KLC2,PSMD8,SLC39A14,NDUFB10,ATP5PD,PSMC5,UCHL1,CYTB,TUBA1A,TUBB4A,KEAP1,COX4I1
